# Detection of arbitrarily-shaped clusters using a neighbor-expanding approach: A case study on murine typhus in South Texas

**DOI:** 10.1186/1476-072X-10-23

**Published:** 2011-03-31

**Authors:** Zhijun Yao, Junmei Tang, F Benjamin Zhan

**Affiliations:** 1 Texas Center for Geographic Information Science, Department of Geography, Texas State University-San Marcos, 601 University Drive, San Marcos, TX, 78666, USA; 2 The Department of Geography and Environmental Systems, University of Maryland, Baltimore County, 1000 Hilltop Circle, Baltimore, MD, 21250, USA; 3 School of Resource and Environmental Science, Wuhan University, Wuhan, 430079, China

## Abstract

**Background:**

Kulldorff's spatial scan statistic has been one of the most widely used statistical methods for automatic detection of clusters in spatial data. One limitation of this method lies in the fact that it has to rely on scan windows with predefined shapes in the search process, and therefore it cannot detect cluster with arbitrary shapes. We employ a new neighbor-expanding approach and introduce two new algorithms to detect cluster with arbitrary shapes in spatial data. These two algorithms are called the maximum-likelihood-first (MLF) algorithm and non-greedy growth (NGG) algorithm. We then compare the performance of these two new algorithms with the spatial scan statistic (SaTScan), Tango's flexibly shaped spatial scan statistic (FlexScan), and Duczmal's simulated annealing (SA) method using two datasets. Furthermore, we utilize the methods to examine clusters of murine typhus cases in South Texas from 1996 to 2006.

**Result:**

When compared with the SaTScan and FlexScan method, the two new algorithms were more flexible and sensitive in detecting the clusters with arbitrary shapes in the test datasets. Clusters detected by the MLF algorithm are statistically more significant than those detected by the NGG algorithm. However, the NGG algorithm appears to be more stable when there are no extreme cluster patterns in the data. For the murine typhus data in South Texas, a large portion of the detected clusters were located in coastal counties where environmental conditions and socioeconomic status of some population groups were at a disadvantage when compared with those in other counties with no clusters of murine typhus cases.

**Conclusion:**

The two new algorithms are effective in detecting the location and boundary of spatial clusters with arbitrary shapes. Additional research is needed to better understand the etiology of the concentration of murine typhus cases in some counties in south Texas.

## Introduction

In recent years, there has been a significant increase in public concern about environmental hazards and disease events [1]. The necessity of identifying the spatial pattern and discovering its underlying causes has culminated in proposing a variety of methods to facilitate this task. Cluster detection methods have been playing an important role in modern epidemic research and public health practice, offering clues to the spatial location of emerging diseases and knowledge of their etiological and pathological causes [1]. A number of spatial statistical methods have been incorporated in cluster detection given the wide adoption of statistical methods since the early 1960s [2]. Many of these methods were developed from statistical indices such as Local Indicators of Spatial Association (LISA) [3] and local G statistic () [4]. These statistical methods were incorporated into some spatial cluster detection methods, such as the Multidirectional Optimal Ecotope-Based Algorithm (AMOEBA) proposed by Aldstadt and Getis [5]. Among these spatial statistic methods, the spatial scan statistic model has been one of the most widely used methods [6,7].

Inspired by the work of Openshaw et al. (1987) [8] and Turnbull et al (1990) [9], Kulldorff (1997) developed a spatial scan statistic that has the capacity to detect clusters of various sizes by placing and moving circular windows across the study area [7]. Rather than specifying the size of a potential cluster *a priori*, this method uses a scan window of varying sizes, corresponding to varying population and varying number of incidents. This method has been applied to many research fields. Examples of these applications include disease pattern analysis [10], criminology [6,11], network [12], as well as ecology and the environment [13]. However, the spatial scan statistic and other similar approaches suffer from some restrictions in practice [14,15]. Although this method can be adopted to include any shape for scan windows [7], it still has limitation in practice due to the predefined geometrical shapes of scan windows [15] which leave a large number of candidate clusters out of the test. It is therefore necessary for researchers to develop methods that can be used to detect clusters with arbitrary shapes.

Recently, many methods and strategies have been proposed to improve the detection of clusters with arbitrary shapes by constructing scanning windows of irregular shapes. Tango and Takahashi (2005) presented a "flexibly shaped spatial scan statistic" (FlexScan) which uses a limited exhaustive search to detect arbitrarily shaped clusters by aggregating its nearest circular neighboring areas [16]. The spatial scan statistic superimposes circular windows on the study area, while FlexScan generates irregularly shaped windows on each area by aggregating its nearest neighboring areas. To reduce the number of arbitrarily shaped scanning windows, Tango and Takahashi [16] limited the length of clusters referring to the relatively small number of areas contained in a scanning window. This method extends the spatial scan statistic to detect irregular shapes but is only applicable for detecting clusters of small or moderate sizes. In addition, the determination of the threshold size of a cluster is very subjective, though Tango and Takahashi (2000) suggested choosing about 10~15 percent of the size of the whole study area as a reasonable number.

One solution to this problem involves setting a constraint to guide the search process so as to reduce the number of candidate scan windows. Patil and Taillie (2004) introduced the concept of "upper level set" and developed an "upper level set scan statistic" [17]. Based on this statistic, a more generalized strategy named minimum spanning tree (also called a cheapest connecting network) was proposed by Assuncao et al (2006) to reduce the number of neighbors to be searched [18]. This method is called a cheapest connecting network or a greedy growth search (GGS) which only absorbs the neighboring areas to maximize the likelihood of a new window. This idea was further improved in the Density-Equalizing Euclidean Minimum Spanning Tree (DEEMST) method proposed by Wieland and her colleagues (2007) [19]. The Minimum Spanning Tree method offers two different functions: in a static minimum spanning tree, the weight refers to the difference of risk rate; in a dynamic minimum spanning tree, the variance of maximum likelihood ratio is taken into account. These methods are similar to GGS as they absorb only the neighboring areas in the search process to maximize the likelihood of a new window. It has the flexibility to start the search from any location in the study area.

GGS cannot avoid the local maximum problem [20]. Many algorithms were adopted or developed to improve the GGS. The genetic algorithm is employed to limit the irregular shape of most potential real clusters [21-23]. Yiannkoulias et al. (2007) presented two approaches to improve the greedy growth search: one is the non-connectivity penalty in order to limit the very irregular cluster shapes and another is the depth limit (*u*) to prevent the generation of large super-clusters from smaller clusters [1]. These approaches will terminate the search in GGS when it fails to increase the likelihood after the predefined steps.

Another famous improvement is a "simulated annealing strategy" proposed by Duczmal and Assuncao (2004). This method is based on graph theory in which nodes present centers of areas, and edges present the geographical relationships among areas [20]. The simulated annealing spatial scan statistic was improved by introducing a non-compactness penalty to reduce the chance that the cluster with extremely irregular shapes would be found [24]. Most of the recent proposed methods try to detect the globally most likely cluster [20,23] and this is critical in cluster detection since the search process of some methods frequently leads to or sticks on the locally most likely clusters.

In this article, we report the development of two algorithms that use a new neighbor-expanding approach based on the assumption that any subset of adjacent areas could make up a potential cluster, and that the shape of this cluster might not be circular or rectangular. These two algorithms are called the maxima -likelihood-first (MLF) algorithm and non-greedy growth (NGG) algorithm. These two algorithms build upon the existing cluster detect techniques, and adopt neighbor-expanding tactics to construct a set of scan windows instead of just using the scan windows in some predefined shapes. Furthermore, the proposed algorithms improve the arbitrarily-shape cluster detection method in avoiding the local maximum problem since the algorithms search for the globally most likely cluster at each step in the search process.

### Two New Algorithms

#### Kulldorff's Spatial Scan Statistic

Because the two algorithms were built upon the spatial scan statistic, it is necessary to review the spatial scan statistic first. Kulldorff's scan statistic method starts from choosing an appropriate probability model of data to compute the likelihood ratio test statistic λ(z) for any scan window z. After identifying primary cluster candidates with the maximum λ(z), a Monte Carlo hypothesis procedure tests the statistical significance and obtains a p-value [25].

In Kulldorff's method [7], one tests the null hypothesis *H*_*0 *_(constant probability for all area) and the alternative hypotheses *H*_*1 *_(the specific area z has a larger probability than outside areas) using either a Bernoulli model or a Poisson model. For a given region **z**, the likelihood function based on the Bernoulli model can be expressed using expression (1):(1)

where, **μ(G) **and **μ(Z) **are the total population of the study area and population in region **Z**; **nG **and **nZ **are the total number of observed cases in the study area and in region **Z**; *p *is the probability that an incident falls in region **Z**, and ***q ***is the probability that an incident falls in the rest of the study area. The likelihood of observing n(Z) in region **z **is given by the function shown below:(2)

Where, . The expected likelihood function has the form as given in expression (3):(3)

Therefore the likelihood ratio λ(z) can be obtained as the quotient through dividing the observed likelihood by expected likelihood:(4)

Kulldorff (1997) also gave the formula to calculate the likelihood ratio based on the Poisson model as shown below [7]:(5)

Once the most likely cluster has been identified, the next step is to test the statistical significance of the detected clusters. To do so, *p*-value, derived from the Monte Carlo simulation, is used to assess the statistical significance of the detected clusters. The Monte Carlo simulation, proposed by Dwass in 1957 [26], was first introduced to cluster detection tests by Turnbull et al. [9]. In a Monte Carlo simulation, a large number of random replications can be generated under a chosen distribution model, conditioned on that the simulated case number will be the same as the real data. In this study, we used the real population counts in each area in the Monte Carlo replication. The disease events in each area are drawn from a non-homogeneous Poisson distribution with mean . The likelihood ratio for each region is calculated using the replica data as well as the real data during the simulation process. Each simulated dataset has a maximum likelihood ratio in the same way as the real data. Then *p*-values can be calculated based on the sorted likelihood ratio of the real data and simulated data. For example, if there are *N *simulated datasets and one real dataset and the total number of datasets will be *N+1*. Within these total datasets, there are *n *simulations having a larger or equal maximum likelihood ratio compared to the one obtained from the real data. That is, the rank of the real data is *n *when we sort the data by their maximum likelihood ratios. The *p-*value for the significant testing in this example will be equal to *n*/(*N*+1). Theoretically, the smaller the p-value, the more likely the cluster is not due to chance. Due to the uncertainty associated with cluster validation, it is suggested that the proposed approach be used as an exploratory rather than a deterministic cluster detection tool.

#### A New Neighbor-expanding Approach

**Figure 1:**
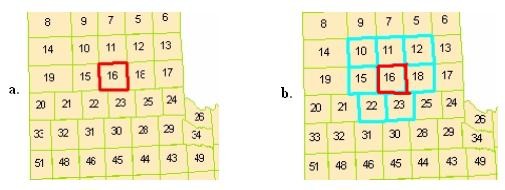
**a) an example map; b) the neighbor areas**. The red color highlights the chosen area and cyan color to highlight the neighbor areas of the chosen area.

A new neighbor-expanding approach is proposed here to detect clusters with arbitrary shapes. Suppose we have a map consisting of a tessellation of component areas. These areas are associated with case numbers and the total population at risk. Two areas are considered as neighbors when they share the same boundary. We assume that a region with any set of connected areas may make up a potential cluster and a cluster may appear in different shapes depending on how many and how aggregated the set of connected areas are. Our goal is to find such clusters with the likelihood ratio in the scan statistic. In the search process, we sweep a large subset of connected areas, constructing a new region at each step by aggregating one of its neighbor areas, until certain thresholds are met or we obtain the expected results. For the sake of simplicity, we use the length to indicate the number of areas that constitute a region. We are always able to get a new region with a higher length ***k+1 ***by combining a ***k ***length region and one of its neighboring areas. One can easily figure out the number of regions with ***k+1 ***length based on a ***k ***length region. If the number of the neighbors around ***k ***length region is ***j***, then one can obtain ***j ***regions at ***k+1 ***length. To clarify, we illustrate this process using an example as shown in Figure 1. In Figure 1, every area is labeled with a number on it. We use a set of numbers to represent the region that is made up of both a region and its neighbors. For example, {16} means a region containing a single area 16 and {16, 18} corresponds to a region consisting of areas 16 and 18.

If we choose {16} as a seed region at first length, we find it highlighted by red color in Figure 1a, we can then get its seven neighbors, areas 10, 11, 12, 15, 18, 22, and 23 (Figure 1b). Thus the seven regions can be obtained at the second length based on region {16}. These seven regions are {10, 16}, {11, 16}, {12, 16}, {15, 16}, {18, 16}, {22, 16}, and {23, 16}. Furthermore, in order to obtain the third length regions, we can choose region {15, 16} and get its neighbor areas: 14, 10, 11, 12, 19, 18, 21, 22, and 23. Now we can get 9 regions at the third length: {14, 15, 16}, {10, 15, 16}, {11, 15, 16}, {12, 15, 16}, {19, 15, 16}, {18, 15, 16}, {21, 15, 16}, {22, 15, 16}, and {23, 15, 16}.

While this search process continues, the number of regions increases exponentially as we aggregate more areas. This process is computationally very intensive. In order to reduce the number of regions, we developed two alternative algorithms for the construction of regions or scan windows: maxima-likelihood-first (MLF) algorithm and non-greedy growth (NGG) algorithm.

##### The Maxima-likelihood-first Algorithm

The principal goal of this algorithm was to direct the new region construction process to obtain a global maximum. This maximum refers to the highest value we were able to obtain by the proposed approach. After analyzing equations (4) and (5), we found that it is hard to determine which of the following factors make the most contribution to the likelihood ratio: the number of cases, population size, or the relationship between them. Thus, there is no clear guidance that could help us construct scan windows which would have the highest likelihood ratios. Rather than construct scan windows randomly, we try to focus on the generation of windows for the most promising clusters. We name this approach as the maximum-likelihood-first (MLF) approach because it always constructs new promising clusters by expanding from the current best candidate, yielding the maximum likelihood ratio.

**Figure 2:**
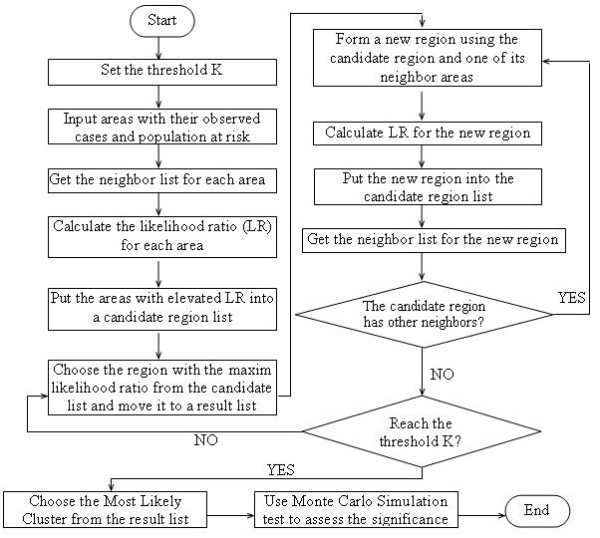
The flowchart of the MLF algorithm

The proposed approach is illustrated in the flowchart in Figure 2. In the initial step of the algorithm, we calculate the Log likelihood ratios (LLRs) for all areas and put the elevated LLRs into a temporary candidate list. After sorting their LLRs in the temporary candidate list, we choose the one with the highest LLR as the candidate region. In the next step, we aggregate the candidate region and one of its neighboring areas to create a new region. A group of new regions are obtained and the LLRs of these new regions are calculated. We put these new regions into the temporary candidate list, sorted the new and old members in the candidate list together again, and choose the one with the new maximum LLR as the new candidate. Unlike the minimum spanning tree algorithm [18], this algorithm expands the neighbors based on multiple seeds in the cluster candidate list. The seed for each neighbor expansion is selected from all the candidates in the temporary candidate list. The procedure is repeated until either the aggregated area covers half of the study area or has half of the total population.

When we detect the cluster using the neighbor-expanding approach described above, it is very likely that the procedure may stick to some areas with high LLRs and unable to search the entire study area. Usually, LLRs of candidate clusters depend on the risk rates of their neighbors [19]. That is, areas with higher risk rates are more likely to have higher LLRs than those with lower risk rates since LLRs of clusters do not vary a lot if they contain the same subset of areas [7]. It means if a candidate cluster overlaps largely with another candidate cluster with a high LLR, it may have a higher LLR than other areas which have not been explored. This observation leads to proposed search procedure to stick with one area and its neighbors if their LLRs increase fast at the beginning and decrease slowly. Therefore, it is necessary to set a threshold to stop the search around a particular area and its neighbors when the LLRs of the newly generated clusters fail to increase in certain steps. This arrangement allows the search to move to other unexplored areas to detect other potential cluster centers. Originally suggested by Yiannakoulias, Rosychuk, and Hodgson (2007) as a depth limit adaptation [1], this idea is incorporated into the MLF algorithm.

As shown in Figure 2, this procedure is repeated until half of the total population or study area is covered. The cluster with the highest LLR is selected as the most likely cluster while the secondary cluster is the cluster having both the second highest LLR with no overlap area with the most likely cluster. Since this approach does not focus on one or some particular areas, it is expected to avoid the local maximum problem.

##### The Non-greedy Growth Algorithm

The non-greedy growth (NGG) algorithm is an improved version of greedy growth algorithm [1]. Several researchers have described how greedy growth approaches perform in searching clusters with irregular shapes [1,24]. The greedy growth search starts with areas having high log likelihood ratio as seed areas for potential clusters. The search is only interested in a neighboring area that has the maximum LLR or has the capability to maximize the LLR when aggregated to form a new potential cluster. Similar to the procedure described above, the greedy growth algorithm joins other areas until a given population size or other thresholds are reached. The same procedure is repeated from other seed areas.

The greedy growth approach sounds tempting, but it has an inherent deficiency in that it does not guarantee to find either the best solution or the global maximum. This method easily falls into the trap of local maximum since it excludes some areas which might potentially form a more promising cluster when they combine with other areas.

To solve this problem, we propose a new algorithm to minimize the impact of the local maximum problem. To distinguish it from traditional greedy growth approaches, we name it "the non-greedy growth algorithm". The algorithm allows not only the neighboring area with the local maximum to be included but also includes many other neighboring areas in the search procedure. Usually the number of newly formed regions relies on the number of candidate regions and the number of neighbors of each region. With this method, we can set a constraint on each of these two numbers control the number of newly formed regions at the next step of the search process. Previous studies suggest that the number of candidate regions increase exponentially, while the number of neighbors of each region does not change dramatically. Therefore, it is more reasonable to set a threshold on the number of candidate regions. Theoretically, if we choose only one candidate and one of its neighbors with the highest LLR each time, this method degrades to the traditional greedy growth search method. The inverse extreme of this approach is the naïve exhaustive approach where no limitation is set.

In the NGG algorithm, we set a threshold (M) on the maximum expected number of new regions at each iteration. Given that threshold and the average number of neighbors, we could easily determine how many candidate regions should be chosen to participate in the aggregation process. There are a few options in the choice of candidate regions. One is to choose M most promising regions, directly from the pool of candidates, or to choose them randomly. In the actual implementation reported in this paper, we used a combination of the two, that is, part of M candidates are from the top regions and the rest are chosen randomly.

**Figure 3:**
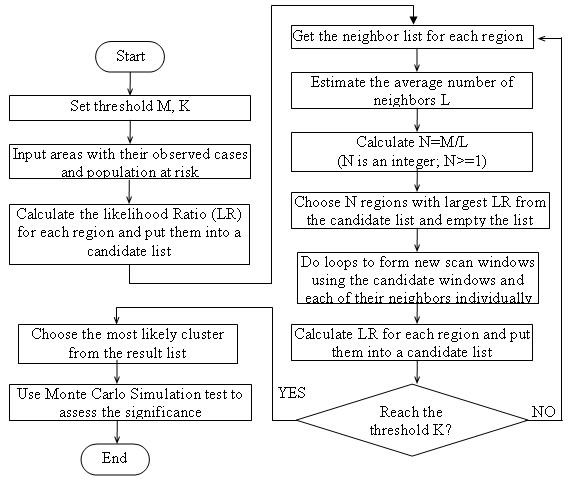
The flowchart of the NGG algorithm

The flowchart showing the NGG algorithm is given in Figure 3. At first, we set a threshold M for the maximum number of potential clusters generated at each step. Then all areas are put into a temporary list and the LLRs of these areas are calculated. In the next step, we calculate the average number of neighbors (L) of each region. The approximate number of candidates (N) for the next iteration is estimated by the preset parameter M and the average number of neighbor L using the equation N = M/L. N areas with the highest LLRs are chosen from the temporary list and the list is emptied afterward. New regions created from the candidates and their neighbors are put into the emptied list. These steps are repeated until either the aggregated area covers half of the study area or has half of the total population. An initial comparison between the MLC and NGG is listed in the Table 1.

**Table 1:** An initial comparison of MLF and NGG

	Advantage	Disadvantage	Favored Situation
MLF	• results might be more significant with higher LLRs • it is faster than NGG when there are few clusters	• it is hard to control when most clusters have relative similar LLRs • only the cluster with the highest LLR is kept into the next search	• data containing few extreme clusters • small number of units

NGG	• the maximum number of candidate cluster is controllable • it is simple to be implemented	• the search procedure will continue until it reaches the criteria	• large number of units

#### Study Case and Data Preparation

**Figure 4:**
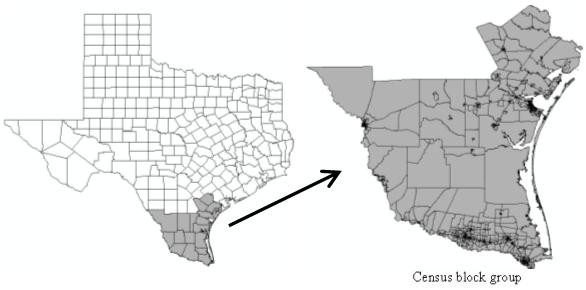
The Study area and the units used in the cluster detection at the scale of Census Block Group

This case study was conducted in the southeast counties in Texas, one of the areas having the most murine typhus cases in the United States (Figure 4). Since the 1970s, the number of murine typhus cases has averaged around 20 per year in this area [27]. Centered at 98°18' W longitude and 27°12' N latitude, our study area includes 18 counties in south Texas with population 1,731,729 in 2000.

The data used in the present study include geographic boundary shapefiles, population data, and disease data issued by the Texas Department of State Health services. In this study, the cluster detection was performed at the census tract block group level and the geographic boundary shapefiles are obtained from Environmental Systems Research Institute (ESRI) website and ESRI Data DVD [28]. There are a total 1,068 census tract block groups and 1,728,393 inhabitants in the study area. The population and socioeconomic data were derived from the 2000 Census Summary File 1 (SF1) and Census Summary File 3 (SF3) [29] and joined to the geographic boundary shapefile to allow for spatial cluster analysis. The disease data used in this study consist of 555 murine typhus cases reported to the Texas Department of State Health Services from 1996 to 2006. Although these cases are reported throughout a year during the period, 44% of cases were found in May, June, and July. The raw disease data were stored in an Excel file, containing the geographical location of cases (latitude and longitude), the onset time of cases (year, month, and day), age, gender, and race of patients, zip code and street name of cases. The disease data have been spatially joined to the boundary file using ArcGIS 9.3.

## Results and Discussions

### Performance Test Using Simulated Data and Benchmark Data

**Figure 5:**
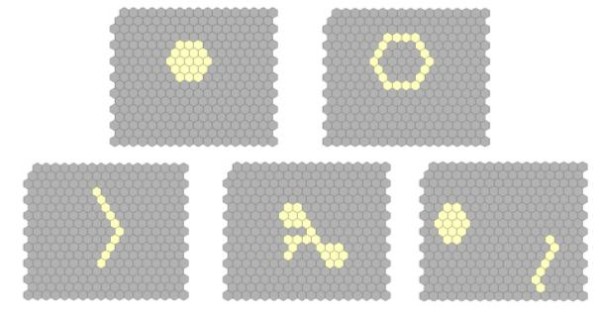
The simulated six cluster patterns for performance tests

We evaluated the performance of the two new algorithms and compared them with the simulated annealing (SA) strategy method, flexible-shape scan statistic (FlexScan), and spatial scan statistic (SaTScan) before we applied the algorithms to the south Texas data. The simulated data consisted of a tessellation of approximately 300 hexagon component areas (Figure 5). These hexagonal areas had the same size. We assumed that populations were homogeneously distributed, and that each hexagonal area had an equal population (1000 persons) subject to disease risk. We assumed areas falling in a synthesized cluster have a high risk rate of 0.5% (5 cases/1000 person) while areas outside have a low risk rate of 0.2% (2 cases/1000 person). The comparisons were based on five different scenarios: a compacted cluster, a ring-shape cluster with regular patterns, an elongated-shape cluster, a strange-shape cluster, and a two-shape cluster with irregular patterns.

**Figure 6:**
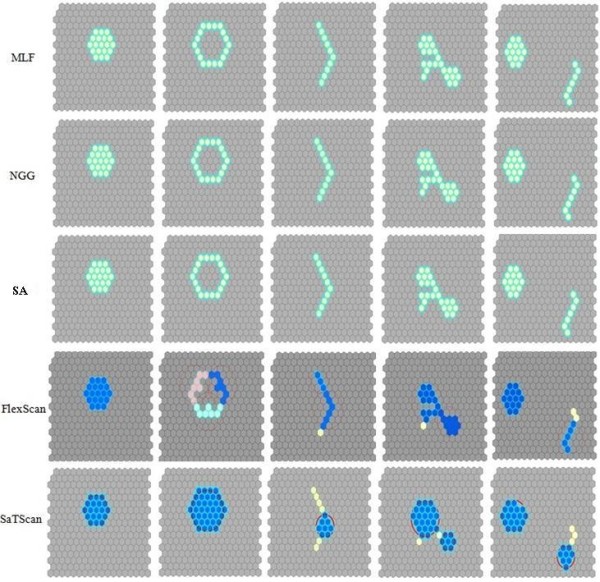
The most likely cluster and secondary cluster detected by MLF, NGG, SA, FlexScan, and SaTScan methods

Figure 6 shows the most likely and secondary clusters detected by the MLF, NGG, SA, FlexScan, and SaTScan, FlexScan methods. Our methods, both MLF and NGG, and SA performed better than the FlexScan and SaTScan methods. Obviously, the SaTScan only performs very well on the compact regular cluster, achieving the same LLR and p-value as other methods (Table 2). However, as the pattern becomes less regular or less compact, the performance of SaTScan becomes unsatisfied. The worst performance was found in the two-cluster pattern, with the largest p-value (0.998) and the smallest LLR value (2.627). The FlexScan method did not perform well in situations involving the ring shape or two-cluster shape with small LLRs (7.165 and 6.599) and large p-values (0.836 and 0.954). The possible reason is that the FlexScan method tries to search for the nearest neighbor; this strategy would trap the search at a location since most of neighbors in the ring and two-cluster patterns are far away from each other. For the extreme irregular shaped patterns, two sub-clusters were detected by the SaTScan with a much less LLR value (9.143) than that of the MLF (32.513). With the two-cluster pattern, the secondary cluster shows much weaker in the SaTScan method with a larger p-value (0.998) and a smaller LLR (2.627). These results indicate that SaTScan and FlexScan are not appropriate in catching clusters with irregular shapes. Actually, these co-existing multiple clusters may create a shadowing effect to each other. There are several methods proposed to solve this problem. For example, Moura et al. (2007) divided the study region into multiple areas before a cluster analysis is performed [30] and Demattei et al. (2007) used a trajectory method in the cluster analysis process to reduce the shadowing effects of multiple clusters to each other [31]. Future research will incorporate these approaches in the methods developed in this study to examine how the proposed methods could be improved to distinguish co-existing multiple clusters.

**Table 2:** The comparison between the MLF method, NNG method, SA method, Tango's FlexScan method and Kulldorff's SaTScan method using the synthesized data

Clusters		Observed #	Expected #	LLR	p-value
	MLF	95	41.646	27.396	0.001
	
Compact shape	NNG	95	41.646	27.396	0.001
	
	SA	95	41.464	27.396	0.001
	
	FlexScan	95	41.646	27.396	0.001
	
	SaTScan	95	41.646	27.396	0.001

	MLF	90	39.273	26.083	0.001
	
	NNG	90	39.273	26.083	0.001
	
Ring shape	SA	90	39.273	26.083	0.001
	
	FlexScan	32	15.273	7.165	0.836
	
	SaTScan	128	80.730	13.756	0.001

	MLF	50	21.010	15.069	0.001
	
	NNG	50	21.010	15.069	0.001
	
Long shape	SA	50	21.010	15.069	0.001
	
	FlexScan	30	12.606	8.866	0.432
	
	SaTScan	28	16.810	3.202	0.993

	MLF	115	51.343	32.513	0.001
	
	NNG	115	51.343	32.513	0.001
	
Extreme shape	SA	115	51.343	32.513	0.001
	
	FlexScan	65	29.020	17.477	0.003
	
		45	20.091	11.877	0.081
	
	SaTScan	86	49.110	12.425	0.001
	
		35	15.630	9.143	0.024

	MLF	70	30.970	19.367	0.001
	
		35	15.485	9.343	0.016
	
	NNG	70	30.970	19.367	0.001
	
		35	15.485	9.343	0.016
	
Two-cluster	SA	70	30.970	19.367	0.001
	
		35	15.485	9.343	0.016
	
	FlexScan	70	30.970	19.367	0.001
	
		25	11.061	6.599	0.954
	
	SaTScan	78	39.820	15.470	0.001
	
		28	17.700	2.627	0.998

**Figure 7:**
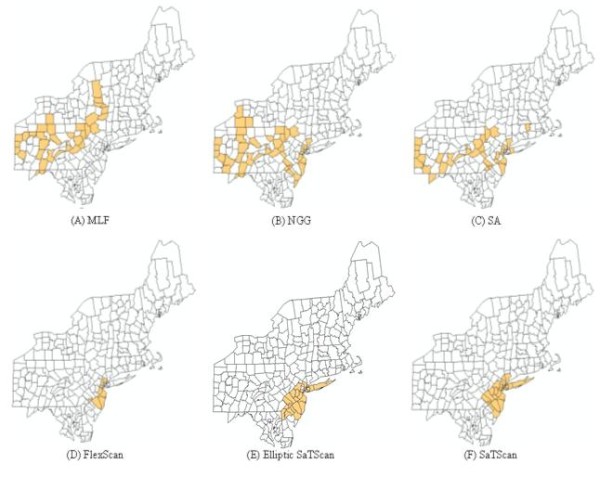
The most likely cluster in the benchmark real disease data detected by MLF, NGG, SA, FlexScan, and SaTScan

A further comparison was performed among these methods using the benchmark real disease data. The data were collected from 11 states and the District of Columbia in the Northeast US from 1988 - 1992, consisting of 58,943 deaths from breast cancer among women. Figure 7 shows the most likely clusters detected by MLF, NGG, SA, FlexScan, and SaTScan methods and Table 3 summarizes these results. For the most detection methods, the most likely clusters had significantly lower p-values (≤0.01) and high LLR values (Table 3). Based on the p-value and LLR values, we conclude that MLF is the most accurate method for detecting clusters with arbitrary shapes, followed in decreasing order by SA, NGG, FlexScan, Elliptic SaTScan, and Circular SaTScan. Meanwhile, it is easy to find that the results detected by the MLF and NGG are less compact than the ones detected by the SaTScan (Figure 7). This indicates that our proposed methods might be less useful than SaTScan in detecting compact clusters.

**Table 3:** A comparison of the MLF method, NNG method, Duczmal's SA method, Tango's FlexScan method, and Kulldorff's SaTScan method using the benchmark data

	MLF	NGG	SA	FlexScan	SaTScan	
					
					Circular	Elliptic
Population	29,535,210					

Total case	58,943					

Observed #	17,002	17,743	15,122	6,980	21,039	15,122

Expected #	14,166	15,383	12,988	6,005	19,734	12,988

LLR	237.24	85.97	227.11	84.11	44.95	44.71

p-value	0.001	0.001	0.001	0.001	0.01	0.001

Note: # means number; LLR means log-likelihood ratio.

### Detection of Cluster with Arbitrary Shapes

**Figure 8:**
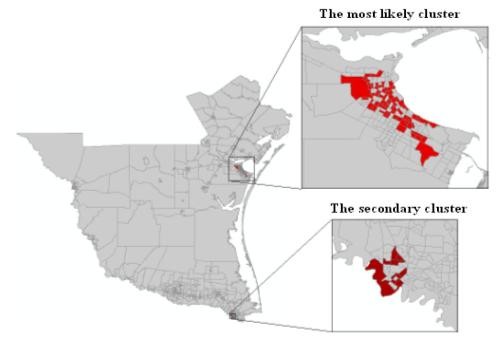
The most likely cluster and the secondary cluster detected by the MLF method at the census block group level

**Figure 9:**
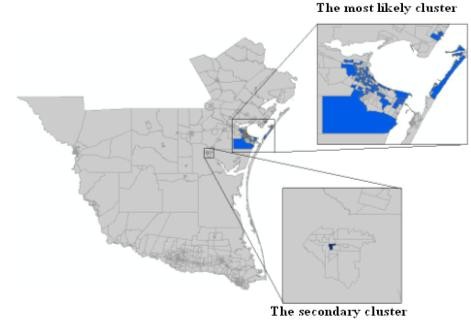
The most likely cluster and the secondary cluster detected by the NGG method at the census block group level

**Figure 10:**
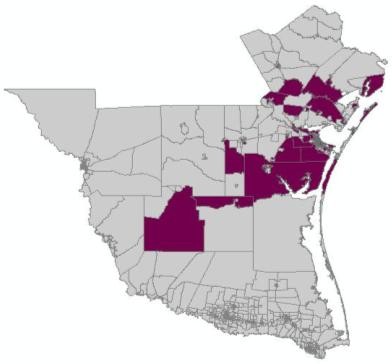
The most likely cluster detected by the SA method at the census block group level

**Figure 11:**
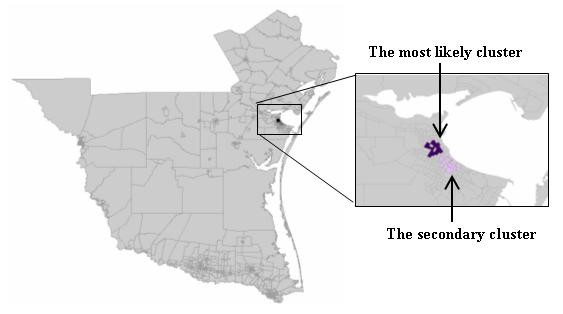
The most likely cluster and the secondary cluster detected by the FlexScan at the census block group level

**Figure 12:**
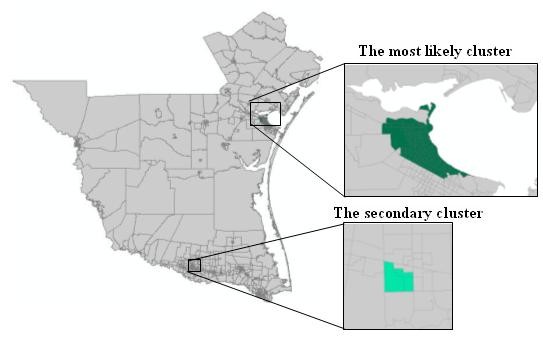
The most likely cluster and the secondary cluster detected by the Elliptic SaTScan method at the census block group level

**Figure 13:**
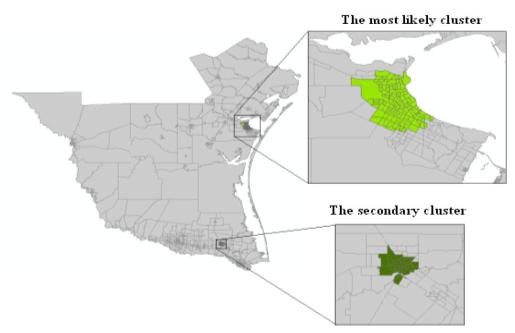
The most likely cluster and the secondary cluster detected by the Circular SaTScan method at the census block group level

The spatial distribution of murine typhus in the south Texas from 1998 - 2008 is identified using the new neighbor-expanding approach developed in this study and traditional SaTScan, FlexScan, and SA methods. The most likely clusters and the secondary clusters detected by the methods are showed in figure 8 (MLF), Figure 9 (NGG), Figure 10 (SA), Figure 11 (FlexScan), Figure 12 (Elliptic SaTScan), and Figure 13 (Circular SaTScan). Both the most likely clusters and the secondary clusters detected by these six methods are highlighted.

As shown in the figures, all the most likely clusters found by the algorithms are significant with a p-vaule of 0.01 and high LLR values. The LLR value of the most likely cluster detected by the MLF algorithm (186.43) and NGG algorithm (197.51) are slightly higher than that of the SA algorithm (177.15) and significantly higher than that of the FlexScan method (42.95) and Circular SaTScan (97.60) (Table 4). The number of most-likely clusters detected by the NGG method (94) is obviously larger than that from the MLF method (71) while the number of secondary clusters detected by the NGG method (1) is much less than that from the MLF method (11). A possible reason for this result is the design of the algorithm itself. Instead of finding the maximum value in the candidate cluster, the NGG algorithm keeps expanding to its neighbors by selecting multiple candidates as seeds for subsequent steps. This procedure will surely lead to a wide distribution of the most likely clusters. Another significant difference found in the NGG algorithm is the shape of detected clusters. Although the distribution of detected clusters is very similar, we still found that the shape of clusters detected by the NGG algorithm (Figure 9) is more irregular than that from the other three algorithms. The potential reason is the same: the algorithm keeps expanding to its neighbors by selecting multiple candidates as seeds for next steps. Since we did not incorporate any penalty function to restrict neighbor expanding, it will influence the direction of the search and the power of the NGG algorithm significantly [24].

**Table 4:** Cluster detection analysis result for Murine Typhus case in the south Texas from 1996 ~ 2000 at the census block group level

	MLF		NGG		FlexScan		SaTScan				SA	
	**Most Likely Cluster**	**Secondary Cluster**	**Most Likely Cluster**	**Secondary Cluster**	**Most Likely Cluster**	**Secondary Cluster**	**Most Likely Cluster**		**Secondary Cluster**		**Most Likely Cluste**	**Secondary Cluster**
									
							**Circular**	**Elliptic**	**Circular**	**Elliptic**		

Population	1,728,393											
Total case	391											
LLR	186.43	9.33	197.51	6.15	42.95	36.95	97.60	124.69	6.67	6.49	177.15	N/A
# of zones	71	11	94	1	16	9	127	121	27	3	164	N/A
Observed #	142	12	167	3	30	25	145	138	2518	6	220	N/A
Expected #	18.96	2.53	26.5	0.15	3.01	2.37	33.54	28.99	6.69	0.87	50.5	N/A
p-value	0.01	0.25	0.01	0.32	0.01	0.01	0.01	0.01	0.42	0.74	0.01	N/A

### Spatial Distribution of Clusters and Socioeconomic Factors

An examination of Figures 8-13 reveals that the presence of the most likely clusters is mainly distributed in the coastal counties, particularly in Nueces County. Caused by two organisms, *Rickettsia typhi *and *R. felis *[32], murine typhus is easily carried and transmitted by small mammals such as mice, domestic cats, and opossums and the associated fleas. Theoretically, the spreading of murine typhus requires a warm and humid environment. This is probably why most of the detected clusters are distributed in the coastal area.

**Figure 14:**
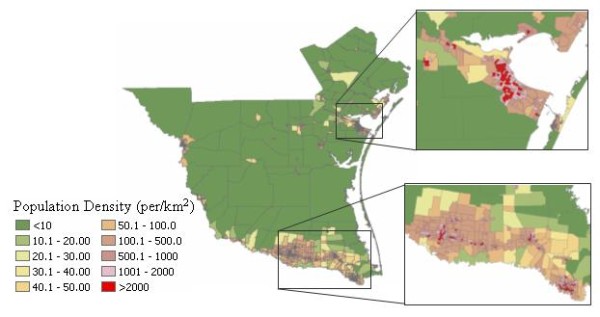
The population density at the census block group level

The distribution of population and related environmental problems might be the reasons responsible for clustering of the cases. Figure 14 is the population density at the census block group level. Of the total 1,068 census block group in the study area, half of them (534) have more than 1,000 persons per square kilometer. Most of these density populated counties are found in the eastern coastal region and in the southern area. The large cities in the southern area are the city of McAllen and Brownsville, the largest city in the eastern coastal region is Corpus Christi. Not surprisingly, these large cities with high population densities are the major seating area of the detected most likely cluster and secondary clusters as revealed by the study. In the MLF method, there are 71 census block groups detected out as the most likely cluster and 66 of them (92.96%) had densities higher than 1,000 persons per square kilometers; 42 of them (59.15%) had densities higher than 2,000 persons per square kilometers (Table 5). A similarly high percentage could be found in FlexScan (100%), Circular SaTScan (91.34%), and Elliptic SaTScan (90.18%).

**Table 5:** Relation between the number of most likely cluster and high density population

		Density > 1000		Density > 2000	
		
	# of cluster	# of cluster	Percent (%)	# of cluster	Percent (%)
MLF	71	66	92.96	42	59.15
NGG	94	77	81.91	44	46.81
SA	164	107	65.24	54	32.93
FlexScan	16	16	100	13	81.25
Elliptic SaTScan	112	101	90.18	59	52.68
Circular SaTScan	127	116	91.34	67	52.76

We can also find the similarity between the distribution of cluster patterns and the environmental factors. Most of reported cases are found in urban areas with very high population densities. Usually, the high density population brings problems, such as increasing amounts of urban garbage and commensal rodents. These will also increase the likely exposure of opossums, a peridomestic animal, to the cat fleas and rickettsial pathogens due to their frequent visiting of human habitation to search for both food and harborage [25]. Moreover, the high population densities also enlarge the number of household pet, which is another common host of cat fleas. Besides the rats and mice, the cat flea is easily switched from the parasitized cats and opossums to other animals of the same size.

To further verify and explain the detected cluster patterns, we collected and analyzed four other socioeconomic factors at both county level and census block group level: median household income, the rate of population with their poverty status below poverty, median house built year, and median value of owner-occupied house units. Nueces County, with the majority of the most likely clusters, has a relative higher median household income ($35,959) and median house value ($70,100) than the average value (median household income $27,026 and median house value $48,467) for all 18 counties. Driven mainly by tourism and the petrochemical industry, the main economic support of Nueces County depends upon its largest coastal city, Corpus Christi, which also drives the development of related commercial real estate and other industries.

**Figure 15:**
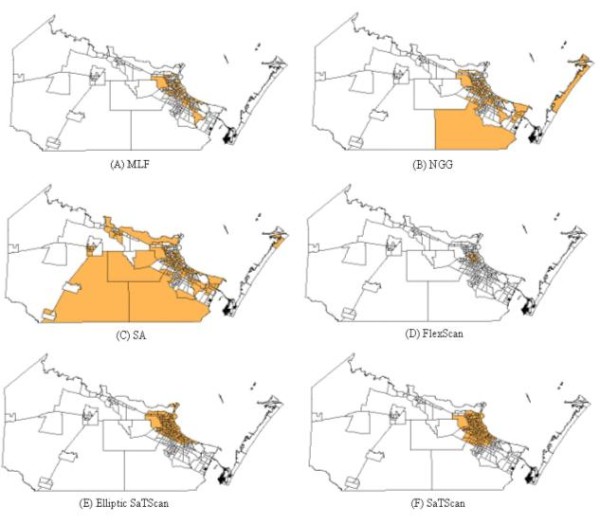
The most likely cluster detected within the Nueces County

For the socioeconomic analysis at the census block group level, we have illustrated the location and distribution of the most likely clusters detected by MLF, NGG, SA, FlexScan, Elliptic SaTScan, and Circular SaTScan within Nueces County (Figure 15) and the associated socioeconomic data (Table 6). Compared to the average value of all block groups within Nueces County, the median household income and house value of the 'clustered' census block groups are obviously lower than those in other block groups. Meanwhile, the poverty rate of this 'hot spot' area is relatively higher than the average poverty rate in all of Nueces County. All these data indicate that the detected cluster patterns agree with the socioeconomic distribution which plays a critical role in the transmission of murine typhus. It is also likely that other information, such as the habitual environment of human and city animals, as well as transmission among people, may be critical in tracking the transmission model. This would be another interesting topic of future research if ancillary data can be obtained in the future.

**Table 6:** Socioeconomic data of the most likely cluster within the Nueces County

Socioeconomic	All block groups	The block groups in the most like cluster detected by					
		
		MLF	NGG	SA	FlexScan	Elliptic SaTScan	Circular SaTScan
Median house income ($)	35,959	31,167	30,469	33,521	26,427	28,419	30,580
Poverty rate (%)	18	21	19	19	24	26	23
Median house built year	1967	1958	1919	1963	1953	1957	1959
Median house value ($)	70,100	58,857	63,074	63,648	49,363	56,033	58,048

## Conclusion

There is an important difference among the performance of traditional SaTScan, FlexScan, SA, and the two algorithms (MLF and NGG) introduced in this paper. Kulldorff's method tries to search the maximum likelihood ratio using a predefined geometrical shape (circle or ellipse) while the FlexScan method would search for the nearest maximum. For most circular-shape clusters, the spatial scan statistic method will promise fast and efficient cluster detection in many applications. That is why this method is popular in providing an initial analysis for most cluster studies. The two new algorithms make it easy to find out the exact location and boundaries of clusters with arbitrary shapes. Moreover, by adopting the idea of global-optimization strategies, the two new algorithms reduce the effects of the local maximum problem by searching for the global maximum of the likelihood ratios at each step.

We compared the detected clusters from the two new algorithms and those from SaTScan, FlexScan, and SA and found the performance of the neighbor-expanding method has been significantly improved in the cluster with arbitrary shapes. However, the computation time of the NGG algorithm was much longer than that of the MLF algorithm. This might be caused by the no-constraint rule when the NGG selects the seed to detect the next level cluster in the search process. Without any penalty on the shape of the result, the NGG allows more detected clusters than the MLF and SA. One possible solution for this problem is to set the degree allowing irregular shape in the detected cluster according to some appropriate criteria, minimizing the occurrence of false clusters. Or we could post-process the entire detected result after cluster analysis to remove the highly irregular ones. But this solution will require more detection time and expert knowledge in selecting an appropriate threshold.

One of the most critical components of environment epidemiology is to estimate the associations between human exposures and health outcomes [33,34]. In order to further understand the etiology of a disease, we need to explore the proximity, frequency, and magnitude of potential environmental hazards and their effects to humans. Obviously, this cluster analysis will help us understand the geographic distribution of murine typhus in Texas. From this cluster analysis, we can easily conclude that the most likely cluster of murine typhus is mostly distributed in warm and humid areas - notably eastern Nueces County along coastal Texas. Moreover, at the census block group level, most of the detected clusters (> 80% or 90%) are in high population density areas (population > 1000 per square kilometer) with lower household incomes and home values. These findings prove that the distribution of murine typhus is controlled by both environmental and socio-economic factors.

The choice of scale/resolution in cluster analysis deserves some attention. In most of case studies, we would prefer to choose a resolution small enough to represent most disease distribution in a relatively homogeneous area. Furthermore, the spatial aggregation of areal data may change the pattern of disease and bring some difficulty in validating the results due to effects of the modifiable areal unit problem (MAUP). A possible solution to this problem involves performing the cluster analyses at different scales of area units to estimate the effects of MAUP and this issue will be addressed in future research. If possible, it would be much better to conduct an analysis of scale effect before conducting a cluster analysis. The choice of scale/resolution for specific cases or specific diseases at different regions should be treated differently. Although there is no specific rule to follow, users of the algorithms should be very familiar with the characteristics of the disease in question as well as the study area before the cluster detection is conducted.

## Competing interests

The authors declare that they have no competing interests.

## Authors' contributions

All authors intensively participated in the study reported in the article. ZY and FBZ conceptualized the study design and analyzed the results. ZY and JT interpreted the results and prepared the initial drafts of the manuscript. All authors participated in the writing of the manuscript and approved the final version.
